# SARS‐CoV‐2 spike protein alleviates atherosclerosis by suppressing macrophage lipid uptake through regulating R‐loop formation on MSR1 mRNA

**DOI:** 10.1002/ctm2.391

**Published:** 2021-09-26

**Authors:** Wenting Zhao, Zhen Wang, Wenjing Chen, Miao Chen, Jie Han, Xiang Yin, Xiaotong Hu, Shuai Wang, Jie Zan, Liangrong Zheng

**Affiliations:** ^1^ Department of Cardiology The First Affiliated Hospital College of Medicine Zhejiang University Hangzhou P. R. China; ^2^ Department of Veterinary Medicine Institute of Preventive Veterinary Sciences Zhejiang University College of Animal Sciences Hangzhou P. R. China; ^3^ Department of Biology Westlake Institute for Advanced Study Hangzhou Zhejiang P. R. China; ^4^ School of Biomedical and Pharmaceutical Sciences Guangdong University of Technology Guangzhou P. R. China

Dear Editor,

Atherosclerosis, as the leading cause of coronary artery disease is one of the major contributors of death globally.^1^, ^2^ SARS‐CoV‐2 causing the novel COVID‐19 respiratory infectious disease is affecting socioeconomic and healthcare systems globally.^3^ Whether SARS‐CoV‐2 infection influences on atherosclerosis development or any virus component can affect or even ameliorate this progress is not clear.

We reanalyzed scRNA‐seq data of COVID‐19 patient blood samples and found SARS‐CoV‐2 infection may affect CD36 and MSR1 expressions which mediate lipid uptake of macrophages contributing to atherosclerosis progression (Supporting information Figure S1). SARS‐CoV‐2 mhain structural proteins, including S (spike), N (nucleocapsid), M (membrane), and E (envelope), were synthesized and transfected (Figure 1A). Combining Q‐PCR and western blot results, we found viral S protein reduces MSR1 expression in macrophages (Figure 1B and C). Lipid uptake analysis shows recombinant S pretreatment or S pseudotype virus infection suppresses macrophage lipid uptake (Figure 1D‐F). And, S incubation or pseudotype virus infection also decreased MSR1 expression in macrophages (Figure 1G). But, MSR1 knockout canceled this inhibitory effect of S or S pseudotype virus on macrophage lipid uptake (Figure 1H and J). Using dual luciferase reporter system, we found S had no significant effect on MSR1 transcription (Figure 1K). RIP assay shown S does not bind to MSR1 mRNA (Figure 1L). Through performing co‐IP and subsequent mass‐spectra analysis, proteins related to metabolism of RNA and mRNA splicing were identified to interact with S (Figure 1M and N). DDX5, involved in multiple steps of RNA metabolism interacts with S protein in macrophages (Figure 1O). In addition, S did not affect DDX5 mRNA or protein expression (Figure 1P and Q). DDX5 overexpression evidently cancels the inhibitory effect of S on MSR1 protein expression (Figure 1R). Overall, these results suggest that S protein suppresses MSR1‐mediated lipid uptake by interacting DDX5.

**FIGURE 1:**
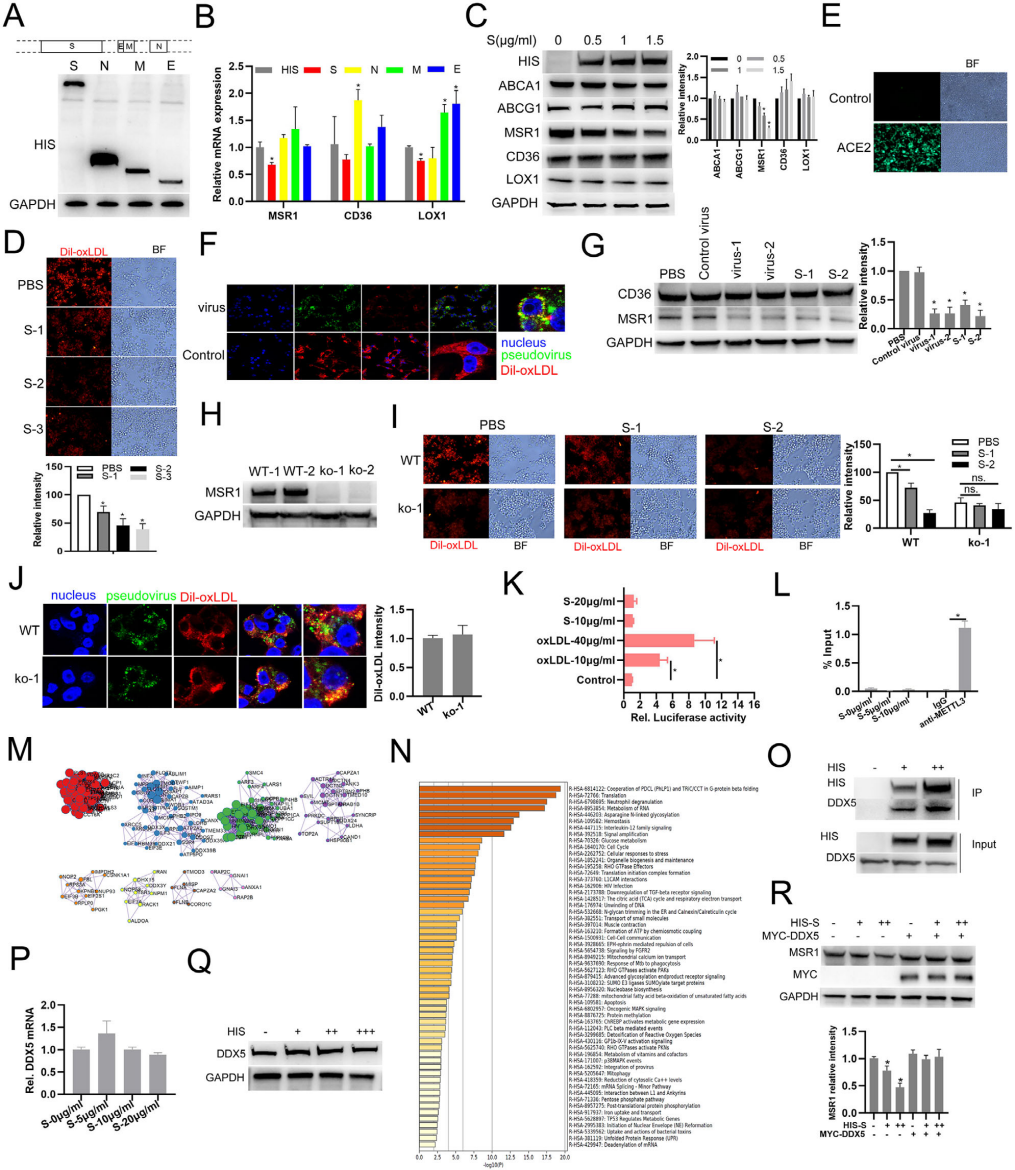
SARS‐CoV‐2 S protein suppresses MSR1‐mediated lipid uptake of macrophage through interacting DDX5. (A) Expression validation of HIS‐tagged SARS‐CoV‐2 main structural proteins including S (spike), N (nucleocapsid), M (membrane), and E (envelope) by Western blot. (B) The indicated genes were detected using qRT‐PCR in macrophages with HIS‐tagged S, N, M, or E plasmids infection. (C) Western blot analysis of the indicated proteins in macrophages with HIS‐tag S plasmids (0.5, 1, or 1.5 μg/mL) infection. (D) Fluorescence analysis of the uptake of Dil‐oxLDL (red) in macrophages incubated with recombinant SARS‐CoV‐2 S protein (S‐1: 1 μg/mL; S‐2: 2 μg/mL; S‐3: 5 μg/mL). (E) Fluorescence analysis of ACE2‐overexpressed 293T cells with SARS‐CoV‐2 pseudotype virus (green) infection for 24 h. (F) Fluorescence analysis of the uptake of Dil‐oxLDL (red) in macrophages with pseudotype virus (green) infection. Nucleus, blue. (G) MSR1 expression was detected using western blot when macrophages were infected with S pseudotype virus or incubated with recombinant S protein. (H) MSR1 knockout THP‐1 cell line was constructed using CRISPER CAS9 technology and validated using western blot (WT‐1: wild type clone‐1; ko‐1: knockout clone‐1; ko‐2: knockout clone‐2). (I) Fluorescence analysis of Dil‐oxLDL (red) uptake in macrophages with PBS or recombinant SARS‐CoV‐2 S protein (S‐1: 1 μg/mL; S‐2: 2 μg/mL). (J) Fluorescence analysis of Dil‐oxLDL (red) uptake in wild type (WT) or MSR1 knockout (ko‐1) macrophages infected with recombinant SARS‐CoV‐2 pseudotype virus (green). Nucleus, blue. (K) Relative luciferase activities were detected in macrophages transfected with PGL3 vectors containing MSR1‐promoter and incubated with or without oxLDL or S for 24 h. (L) RNA immunoprecipitation analysis of the association between S and MSR1 mRNA in macrophages with anti‐HIS antibody (anti‐METTL3 antibody as a positive control). (M) Cluster analysis of proteins potentially interacted with S using Metascape. (N) GO enrichment analysis of proteins potentially interacted with S protein. (O) Co‐IP analysis of S (+, ++: 5, 10 μg) interacting with DDX5 in macrophages incubated with HIS‐tagged recombinant viral S protein by anti‐HIS antibody. (P) qRT‐PCR analysis of DDX5 mRNA expression in macrophages incubated with HIS‐tagged recombinant viral S protein. (Q) Western blot analysis of DDX5 protein expression in macrophages incubated with HIS‐tagged recombinant viral S protein (+, ++, +++: 5, 10, 20 μg). (R) Western blot analysis of MSR1 protein expression in macrophages transfected with MYC‐tagged DDX5 plasmids and incubated with HIS‐tagged recombinant viral S protein (+, ++: 5, 10 μg).^*^ *P* < .05

To explore the underlying mechanism involved in, we first examined the effect of S on the subcellular distribution of DDX5 in macrophages and found that S promotes DDX5 cytoplasmic location and inhibits its nuclear location (Figure 2A and B). Next, we constructed HIS‐tagged S1 and S2, which are cleaved products of S protein,^4^ and MYC‐tagged DDX5. Co‐IP analysis showed that both full length viral S protein and S2 are coprecipitated with DDX5 suggesting that S2 is responsible for interacting with DDX5 in cytoplasm (Figure 2C). Subsequently, we synthesized plasmids expressing S1 or S2 with a cell‐penetrating signal from tat protein (GRKKRRQRRR)^5^ fused to the C‐termination enhancing the efficacy of S1 and S2 entering into cells. Macrophages were incubated with S1 or S2 protein and Western blot analysis showed that S2 but not S1 incubation indeed decreases MSR1 expression (Figure 2D and E). Rescue experiments shows overexpression of DDX5 prominently abolishes the inhibitory effect of viral S2 protein on MSR1 expression (Figure 2F). Domain mapping analysis shows that N‐terminal domain of DDX5 is responsible for interaction with S protein (Figure 2G). Our previous study found that DDX5 regulates N6‐methyladenosine (m6A) modification on MSR1 mRNA by interacting with methyltransferase‐like 3 (METTL3)^6^ and we found overexpression of DDX5 or METTL3 decreases or increases m6A modification on MSR1 mRNA respectively (Figure 2H). Considering that m6A participates in the formation of RNA:DNA hybrids (R‐loop), which controls a series of biological processes, including gene transcription,^7^, ^8^ and DDX5 plays crucial roles in R‐loop resolution to promote gene transcription.^9^ Through using used S9.6 antibody which specifically recognizes and precipitates R‐loop structure,^10^ we demonstrated that DDX5 cooperates with METTL3 to regulate MSR1 mRNA transcription through modifying R‐loop resolution (Figure 2I‐P). Overall, these results indicate that DDX5 N‐terminal domain interacts with S2 region of S protein which is responsible for inhibition of DDX5‐mediated MSR1 expression and DDX5 cooperates with METTL3 to regulate MSR1 mRNA transcription through modifying R‐loop resolution.

**FIGURE 2:**
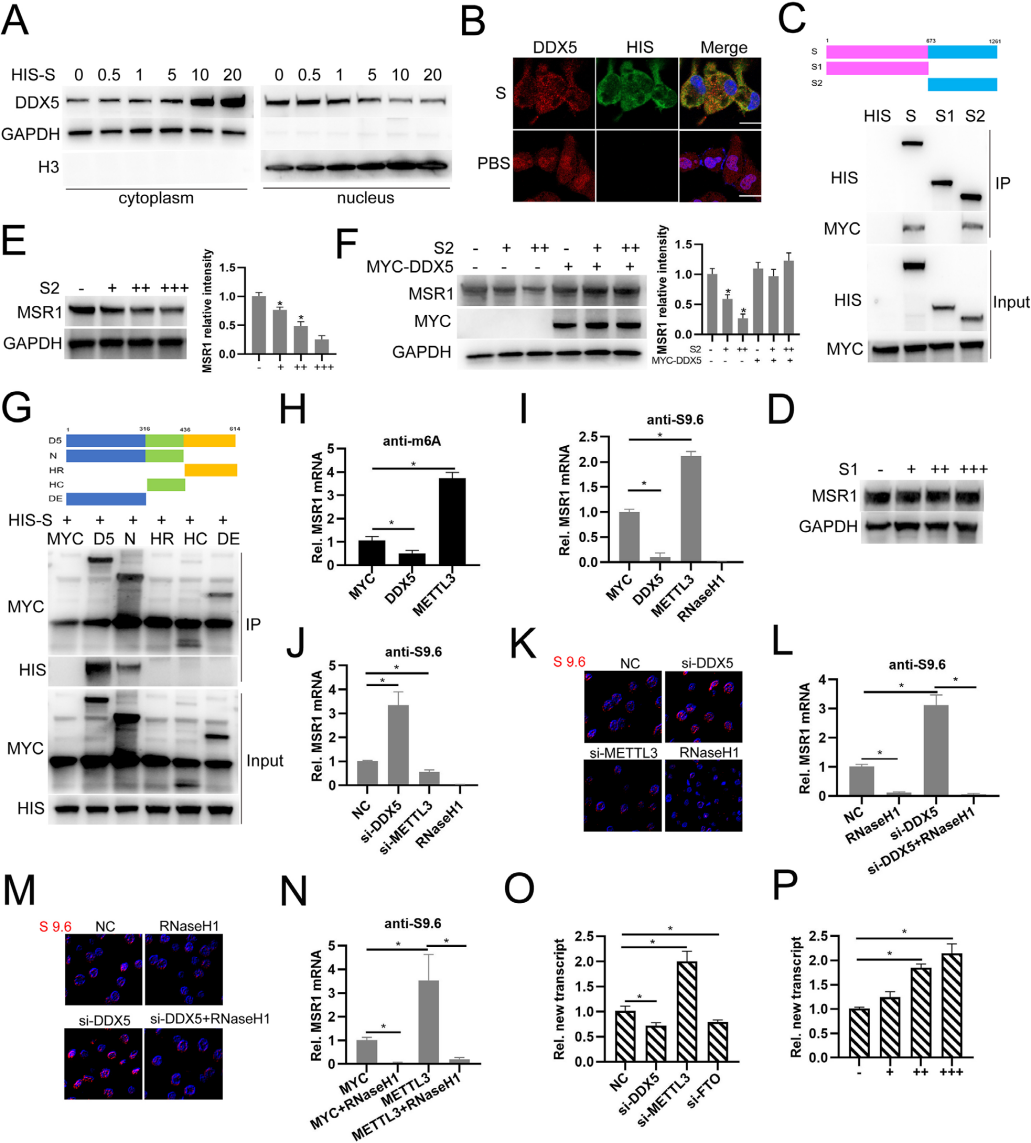
DDX5 N terminal domain interacts with S2 region of S protein which mediates MSR1 expression inhibition and DDX5 cooperates with METTL3 to regulating MSR1 mRNA transcription through modifying R‐loop resolution. (A) Western blot detection of DDX5 protein in the cytoplasmic or the nuclear fractions of macrophages incubated with HIS‐tagged recombinant viral S protein (GAPDH as cytoplasmic maker; Histone‐H3 as nuclear maker). (B) Fluorescence analysis of the subcellular distribution of DDX5 (red) in macrophages incubated with recombinant viral S protein (green) or PBS. Nucleus, blue. (C) Domain mapping analysis of the interaction between MYC‐tagged DDX5 protein and HIS‐tagged viral S protein S, S1 region or S2 region by Co‐IP. (D‐E) Western blot analysis of MSR1 expression in macrophages incubated with HIS‐tagged recombinant viral S1 protein (D) or S2 protein (E). (F) Western blot analysis of MSR1 expression in macrophages transfected with MYC‐tagged DDX5 plasmids and incubated with HIS‐tagged recombinant viral S2 protein. (G) Domain mapping analysis of the interaction between MYC‐tagged DDX5, *N*‐terminal domain (N), p68HR domain (HR), HELIC domain (HC), DEXD domain (DE), and HIS‐tagged recombinant viral S2 protein in 293T cells. (H) MeRIP‐qRTPCR analysis of MSR1 mRNA expression in macrophages transfected with MYC, MYC‐DDX5, or MYC‐METTL3 plasmids. (I) DRIP‐qRTPCR analysis of MSR1 mRNA expression in macrophages transfected with MYC, MYC‐DDX5, or MYC‐METTL3 plasmids. (J) DRIP‐qRTPCR analysis of MSR1 mRNA expression in macrophages transfected with control siRNA (NC), siRNA‐DDX5, or siRNA‐METTL3. (K) Fluorescence analysis of the subcellular distribution of R‐loop foci in macrophages transfected with NC, si‐DDX5, or si‐METTL3. (L) DRIP‐qRTPCR analysis of MSR1 mRNA in macrophages transfected with NC or si‐DDX5 with RNaseH1 treatment or not. (M) Fluorescence analysis of the subcellular distribution of R‐loop foci in macrophages transfected with NC or si‐DDX5 with RNaseH1 treatment or not. (N) DRIP‐qRTPCR analysis of MSR1 mRNA in macrophages transfected with MYC, or MYC‐METTL3 plasmid with RNaseH1 treatment or not. (O) qRTPCR analysis of MSR1 new transcript in macrophages transfected with NC, si‐DDX5, si‐METTL3, or si‐FTO siRNAs. (P) qRTPCR analysis of MSR1 new transcript in macrophages treated with RNaseH1. *P < .05

Furthermore, we identified viral S or S2 protein treatment significantly increased m6A modifications, R‐loop formation, and R‐loop foci on MSR1 mRNA (Figure 3A‐C). Co‐IP analysis showed that viral S or S2 treatment indeed decreases DDX5 binding to METTL3 (Figure 3D). RIP analysis showed that knockdown of DDX5 further strengthens viral S or S2 protein‐mediated m6A modification or R‐loop formation on MSR1 mRNA, but knockdown of METTL3 almost abolishes (Figure 3E and F). Finally, we constructed atherosclerotic mice model and injected with recombinant S2 protein (Supporting information Figure S2). Immunofluorescent staining of aortic plaque area showed that DDX5 expression in macrophages is significantly higher in atherosclerotic mice than that in the control mice (Figure 3G). And, HIS‐tagged viral S2 protein could be identified in the plaque of recombinant S2 protein injected in atherosclerotic mice (Figure 3H). Moreover, en face aorta and aortic HE analysis indicated that compared to the PBS injected atherosclerotic mice, the lesion area is smaller in recombinant viral S2 protein injection group (Figure 3I‐K) suggesting S2 protein injection alleviates atherosclerosis progression. We also found MSR1 expression decreases and DDX5 nuclear location distribution is reduced after S2 treatment (Figure 3L and M). Finally, we sorted F4/80+ macrophages from aorta samples of PBS or viral S2 protein‐infected atherosclerotic mice and detected the levels of m6A and R‐loop foci by immunofluorescent staining and found viral S2 protein treatment significantly promotes colocalization of m6A and R‐loop in nucleus (Figure 3N). Overall, these results suggest that SARS‐CoV‐2 S2 protein could induce m6A modification or R‐loop formation on MSR1 mRNA and relieve atherosclerosis development.

**FIGURE 3:**
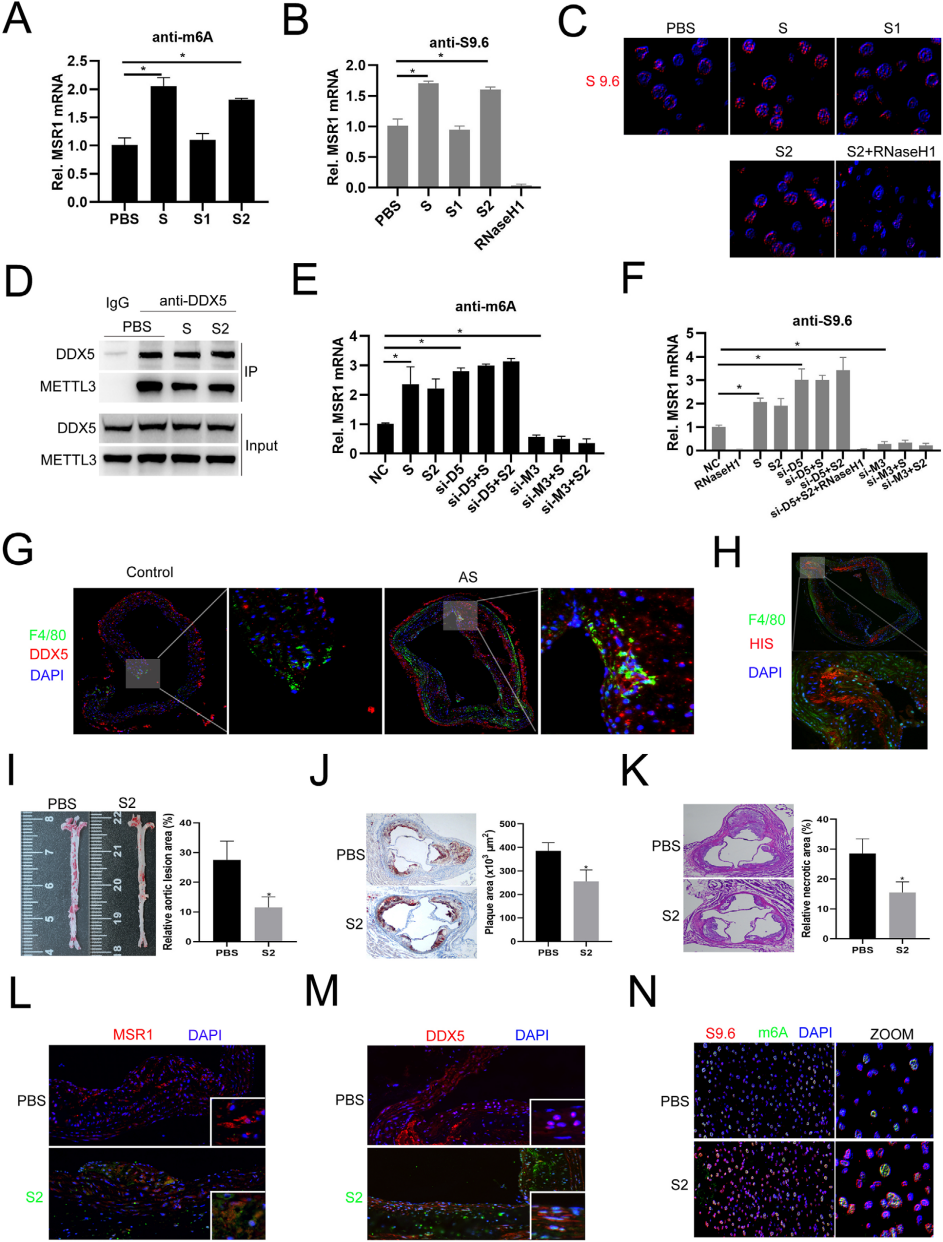
SARS‐CoV‐2 S hinders the association between DDX5 and METTL3 to decrease R‐loop formation on MSR1 mRNA and S2 alleviates atherosclerosis progression in ApoE‐/‐ mice. (A) MeRIP‐QPCR analysis of MSR1 mRNA in macrophages incubated with HIS‐tagged recombinant viral S, S1, S2 protein or PBS. (B) DRIP‐QPCR analysis of MSR1 mRNA in macrophages incubated with HIS‐tagged recombinant viral S, S1, S2 protein or PBS. (C) Fluorescence analysis of the subcellular distribution of R‐loop foci in macrophages incubated with HIS‐tagged recombinant viral S, S1, S2 protein or PBS. (D) Coimmunoprecipitation analysis of the association between DDX5 and recombinant viral S or S2 protein in macrophages incubated with recombinant viral S or S2 protein. (E) MeRIP‐qRTPCR analysis of MSR1 mRNA in macrophages transfected with NC, si‐DDX5, or si‐METTL3 siRNA or incubated with recombinant viral S, or S2 protein, or transfected with si‐DDX5 or si‐METTL3 before incubated with recombinant S or S2 protein. (F) DRIP‐QPCR analysis of MSR1 mRNA in macrophages transfected with NC, si‐DDX5 or si‐METTL3 siRNA or incubated with recombinant viral S, or S2 protein, or transfected with si‐DDX5 or si‐METTL3 before incubated with recombinant S or S2 protein. (G) Immunofluorescence analysis of F4/80 (green) and DDX5 (red) expression in the normal aortic regions of normal mice (Control) or atherosclerotic plaque regions of AS model mice, respectively. (H) Immunofluorescence analysis of F4/80 (green) and HIS‐tagged viral S2 (red) expression in plaque regions of AS model mice. (I) Oil red O staining images and quantification in the aorta en face lesion (n = 10 for each group). (J) Oil red O staining images and quantification in the aortic root lesion area (n = 10 for each group). (K) H & E staining images and quantification in the aortic necrotic core area (n = 10 per group). (L) Immunofluorescence analysis of MSR1 (red) expression in the plaque regions of AS model mice (with or without S2 [green] treatment). (M) Immunofluorescence analysis of DDX5 (red) location in the plaque regions of AS model mice (with or without S2 [green] treatment). (N) Immunofluorescence analysis of the colocalization of m6A (Green) and S9.6 (Red) in F4/80 positive cells sorted from PBS and S2 group mice. *P < .05

In conclusions, we for the first time demonstrate that SARS‐CoV‐2 S protein associates with DDX5 to decrease MSR1 expression suppressing macrophage lipid uptake to alleviate atherosclerosis. Although the relationship between viral infection and atherosclerosis is still unclear and needs more attention in the future, our study sheds a new light on COVID‐19 and atherosclerosis. However, whether other helicases also involve in regulating lipid uptake of macrophages, what is the function of nuclear viral S protein and whether it directly affects MSR1 gene transcription need to be further investigated.

## CONFLICT OF INTEREST

All authors declare no conflict of interest.

## FUNDING

This study was supported by National Key R&D Program of China (Grant no. 2016YFC1301003), and a grant from the Department of Science and Technology, Zhejiang Province (Grant no. LGF19H020011), a Grant from the Department of Science and Technology, Zhejiang Province (Grant no. Z16H020001) and the Natural Science Foundation of China (Grant no. 81873484), People's Republic of China.

## AUTHOR CONTRIBUTIONS

Z.L.R., Z.J., and W.S. conceived the research and participated in the study design. C.M., H.J., Y.X., and H.X.T. assisted in collecting the data. Z.W.T., W.Z., and C.W.J. analyzed the data and drafted the manuscript. All authors read and approved the final manuscript.

## Supporting information

Supporting Information
