# Supplementary material for: SARS‐CoV‐2 spike protein alleviates atherosclerosis by suppressing macrophage lipid uptake through regulating R‐loop formation on MSR1 mRNA
Source: Clin Transl Med. 2021 Sep 26;11(9):e391. doi: 10.1002/ctm2.391 (PMC8473477; doi:10.1002/ctm2.391)
Supplement: Supplementary file 1 — Supporting Information [file CTM2-11-e391-s001.docx]

**SUPPLEMENTARY INFORMATION**

**SARS-CoV-2 Spike protein alleviates atherosclerosis by suppressing macrophage** **lipid uptake through regulating R-loop formation on MSR1 mRNA**

The ethical of this work was reviewed by the ethics committee of the First Affiliated Hospital, College of Medicine, Zhejiang University. More detailed descriptions of the materials and methods were shown in Supplemental Materials.

1. **Materials and Methods**

**1.1 Analysis of lipid-related genes expression levels in PBMCs**

Single cell RNA sequence data from peripheral blood mononuclear cells (PBMCs) from COVID-19 patients (GSE150861), and healthy donors (GSE4557334) were used for cell filtration, clustering analysis, and uniform manifold approximation and projection (UMAP) with R package Seurat as described in the vignettes (https://satijalab.org/seurat/vignettes.html). Cells expressing <200 or >2500 transcripts were filtered out. Next, the data were log‐normalized and highly variable genes were selected using the FindVariableFeatures function and downstream procedures were performed using the ScaleData and runPCA function. Then, FindClusters function was performed to cluster cells and UMAP was used to visualize clusters. Visualization of lipid-related genes expression were shown with violin plot. The Find All Markers function (min.pct = 0.25, logfc. threshold = 0.25) was used to identify marker genes.

**1.2 Cell culture and siRNA transfection**

Macrophages were induced from human primary monocytes, which were separated from human peripheral blood using lymphocyte separation medium (Sigma, USA). Monocytes were cultured in dishes with completed culture medium (RPMI1640, Gibco, USA) supplemented with 10% FBS (Gibco, USA) and 100 ng/ml granulocyte-macrophage colony-stimulating factor (GM-CSF, R&D, USA). Completed medium was changed every other day. After seven days, cells were seeded in six-well plates with 2 ml of culture medium for later experiments. The siRNA transfections were performed as follows: Cells were cultured in serum-free medium for 30 min before transfection. A total of 2 μl of siRNA (50 nM, Ruibo, China) and 3.3 μl of RNAiMAX reagent (Invitrogen, USA) were mixed, incubated for 10 min, and added to each well. Culture medium was changed with fetal calf serum containing culture medium six hours later. The plates were allowed to incubate at 37 °C for 24 h for subsequent detection.

**1.3 RNA extraction and real-time qPCR analysis**

Total RNA was extracted using an RNA prep Pure Cell/Bacteria Kit (Tiangen, China), and then reverse-transcribed into cDNA using PrimeScript RT Master Mix (Takara, Japan). SYBR Premix Ex TaqII (Takara, Japan) kit was used for qRT-PCR detection with ABI PRISM 7500 Detection System (ABI, USA). Values were normalized by GAPDH. The primers used were as follows: GAPDH, 5′-GGAGCGAGATCCCTCCAAAAT-3′ and 5′-GGCTGTTGTCATACTTCTCATGG-3′; MSR1, 5′-TGAACGAGAGGATGCTGACTG-3′ and 5′-TGTCATTGAACGTGCGTCAAA-3′; CD36, 5′-ATGGGCTGTGATCGGAACTG-3′ and 5′-GTCTTCCCAATAAGCATGTCTCC-3′; LOX-1, 5′-CTGGATTGGATTGCATCGGAA-3′ and 5′-CAGCTCCGTCTTGAAGGTATG-3′; DDX5, 5′-AGAGAGGCGATGGGCCTATTT-3′ and 5′- CTTCAAGCGACATGCTCTACAA-3′. All samples were quantitated using the comparative CT method and normalized to GAPDH.

**1.4 Western blot and Mass spectrometry**

Cells were lysed using cell lysis buffer (CST, USA) to extract the total protein. The nuclear fraction and the cytoplasmic fraction were isolated using the nuclear/cytoplasmic protein extraction kit (Beyotime, China). Total protein concentration was detected using the BCA protein assay kit (Thermo, USA). Protein samples were separated on a 10% SDS-PAGE gel and then transferred to PVDF membranes (Millipore, USA). The membranes were blocked with 5% non-fat milk for 1 h at room temperature and then incubated overnight at 4 °C with the following primary antibodies: rabbit anti-GAPDH (1:1000, Sangon, China), mouse anti-DDX5 (1:1000, abcam, USA), rabbit anti-MSR1 (1:1000, Abcam, USA), rabbit anti-CD36 (1:1000, Cell Signaling Technology, USA), rabbit anti-LOX1 (1:1000, Abcam, USA), mouse anti-ABCA1 (1:1000, Abcam, USA), rabbit anti-ABCG1 (1:1000, Abcam, USA), rabbit anti-METTL3 (1:1000, Abcam, USA), rabbit anti-MYC (1:2000, abcam, USA), rabbit anti-6×HIS (1:2000, abcam, USA), rabbit anti-histone H3 (1:2000, abcam, USA). After incubation with specific secondary antibodies (1:10000, Kangwei, China), membranes were developed using the ECL kit (Pierce, USA). For mass spectrometry analysis, protein extracts were incubated with HIS-tag protein purification beads (Solarbio, China) on a rotating wheel at 4°C. The immune-precipitation proteins were boiled and subjected to SDS-PAGE. Gels were stained with the Fast silver stain kit (Beyotime, China). Proteins specifically interacting with S were identified using tandem mass spectrometry as Beijing Protein Innovation (ACQUITYTMUPLC-QTOF, China).

**1.5 Luciferase reporter assay**

Genomic DNA was extracted using the Genomic DNA Extraction kit (TianGen, China) from macrophages. MSR1 promoter sequence was amplified using PCR. The PCR products were gel-purified (PureLink PCR Purification Kit, ThermoFisher, USA) and then inserted into a pGL3-basic luciferase reporter vector (Promega, USA) between HindIII and SacI restriction sites. Then, macrophages were transfected with 750 ng of pGL3-basic/MSR1promoter plasmids using jetPET macrophage transfection reagent (Polyplus, France) with or without oxLDL (Yiyuan, Guangzhou, China) or recombinant S protein (Yiqiaoshenzhou, China). In addition, 20 ng of pRL-TK (renilla luciferase control vector from Promega) plasmids was also co-transfected. After transfection, oxLDL or recombinant S protein was added (indicated concentration). The luciferase activity was detected 48 h after transfection using the Dual Luciferase Reporter system (Promega) by normalizing to renilla luciferase activity.

**1.6 Co-IP (Co- immunoprecipitation) assay and MS (tandem mass spectrometry) analysis**

Macrophages were lysed and incubated with recombinant SARS-CoV-2 S protein for 24 h. After incubated with anti-6×HIS antibody for another 2h, the bound proteins were subjected to washing three times for a total of 30 min and then eluted by boiling for 5 min in the loading buffer. Immunocomplexes were analyzed by SDS-PAGE and then incubated with anti-DDX5 or anti-HIS antibody. Each sample was resuspended in loading buffer (0.1% formic acid, 0.03% trifluoroacetic acid, 1% acetonitrile) and analyzed independently by reverse-phase liquid chromatography coupled with tandem mass spectrometry (LC-MS/MS) using ACQUITYTM UPLC-QTOF analysis platform at Beijing Protein Innovation (Beijing, China).

**1.7 Me-RIP (methylated RNA-binding protein immunoprecipitation) and DRIP (DNA/RNA immunoprecipitation)**

Me-RIP/DRIP was conducted using the Magna RIP kit (Millipore, USA). In brief, the cells were harvested after washing twice with 1× PBS and then lysed with RIP lysis buffer. For DRIP assay, cells were lysed with RNase III/T1 (Ambion, USA) to digest long double-stranded RNA and non-hybridized RNA region in DNA:RNA bybrid. The supernatant was incubated with antibodies against m6A (Synaptic System, German) or DNA:RNA hybrid S9.6 antibody (Abcam, USA) overnight at 4 °C. Then, 50 μl A/G magnetic beads were added to the supernatant and incubated for 6 h. After immobilizing the magnetic bead bound complexes with a magnetic separator (Millipore USA), supernatants were used to extract RNA with PCA (phenol: chloroform: isoamyl alcohol) reagent at a ratio of 125:24:1 (Aladdin, USA). A cDNA synthesis kit (ABI USA) was used to synthesize the first cDNA strand from mRNA. Finally, qRT-PCR was performed using SYBR green master mix (Takara, JAPAN). Gene expression was normalized by comparison to levels of GAPDH gene expression.

**1.8 Immunofluorescence**

Cells were seeded in 35-mm glass bottom dishes, fixed and permeabilized at 4°C for 30 min. After incubation with anti-DDX5 antibody, anti-HIS antibody, anti-m6A antibody or anti-DNA/RNA hybrid S9.6 antibody at 4°C overnight, the cells were washed with PBS twice and stained with goat-anti-rabbit FITC-labelled IgG or goat-anti-mouse rhodamine-labelled IgG (abcam, USA) at 4°C for 2 h, followed by DAPI staining. The cells were viewed using a Zeiss Confocal Microscope Imaging System (Carl Zeiss, Germany).

**1.9 Plasmids construction and protein purification**

The full length sequence of 6xHIS-SARS-CoV-2 S and DDX5 was synthesized directly (Sangon, China) and cloned into pCMV vector or pCMV-MYC vector, respectively. DDX5 N terminal domain (N), p68HR domain (HR), HELIC domain (HC) and DEXD domain (DE) constructs were generated by PCR-based amplification using the wild-type pCMV-DDX5 as the template. All constructs were confirmed by sequencing. Plasmids were transiently transfected into 293T cells using jetprime DNA transfection reagents (Polyplus, France) according to the manufacturer’s instructions.

**1.10 Protein purification**

The pronucleus expression plasmids pET-28a containing cDNAs of different SARS-CoV-2 S parts fusing to N-terminal tagged 6×Histidine were synthesized directly (Sangon, China), and then transformed into transetta (DE3) chemically competent cells (Transgene Biotech, USA). Transformed cells were grown at 37°C to a density of 0.6 to 0.8 at OD600 (optical density at 600 nm) and induced with 0.5 mM isopropyl-β-D-thiogalactopyranoside at 25°C for 6 hours. The cells were collected and resuspended in lysis buffer [25 mM tris-HCl (pH 7.5), 150 mM NaCl, 5% glycerol, and 20 mM imidazole]. The cells were lysed and protein supernatants were allowed to flow through a Ni-NTA Sefinose Column (Sangon, China). The columns were washed with lysis buffer and 100 mM imidazole. The proteins were eluted with elution buffer [25 mM tris-HCl (pH 7.5), 150 mM NaCl, 10% glycerol, and 300 mM imidazole]. To remove imidazole, the eluted fraction was dialyzed in dialysis buffer [25 mM tris-HCl (pH 7.5), 150 mM NaCl, and 10% glycerol] and stored at −80°C.

**1.11 Establishment of MSR1 knockout THP-1 cells**

MSR1 knockout THP-1 cells (MSR1-KO THP1 cells) were established using the CRISPR/Cas9 genome editing system. The MSR1-KO lentivirus-based CRISPR plasmid (designated as the MSR1-KO plasmid) was also a kind gift from Prof. Li. The single guide RNA (sgRNA) sequence used to disrupt the MSR1 gene was 5’-ACAT GGAAGCCAAC CTCA-3’. This plasmid was packaged according to an established protocol [54]. THP-1 cells were seeded into a 6-well plate at a density of 5 × 10^5^ cells, and subsequently infected with the MSR1 KO plasmid-encoding lentivirus for 24 h and cultured in RPMI 1640 medium supplemented with 10% FBS. Forty-eight hours after infection, cells were selected in a culture medium containing 1 μg/mL puromycin for 2 weeks. Surviving cells were then trypsinized (Gibco) to obtain single cells that were seeded into 96-well plates at 1 cell per well. Clones derived from single cells were selected, and Western blot was used to identify the efficiency of genome editing after a clone expansion period. Clones with no detectable caspase-3 signal were selected for further study.

**1.12 SARS-CoV-2 pseudotype virus infection**

The SARS-CoV-2 virus pseudotype was purchased from Paizhen Biotech Co. (Guangzhou, China). HEK293T cells transfected with pcDNA3.1-ACE2 plasmid or macrophages were seeded into 24-well plates and infected with the SARS-CoV-2 pseudotype virus (MOI=5) for 24 h.

**1.13 Lipid uptake analysis**

After macrophages were pretreated with recombinant S protein or infected with SARS-CoV-2 pseudotype virus (MOI=5), cells were treated with oxLDL (10 μg/ml) for 24h. Then, cells were subsequently incubated with Dil labeled-human oxidized low density lipoprotein (Dil-oxLDL) (10 ng/ml, Yiyuan, Guangzhou, China) for 30 min. Next, cells were washed twice with ice-cold PBS and observed using a Zeiss Confocal Microscope Imaging System (Carl Zeiss, Germany).

**1.14 Flow Cytometry**

Briefly, aorta samples of mice were first cleaned of fatty tissues and digested with aorta dissociation enzyme stock solutions (125 U/ml collagenase type XI, 60 U/ml hyaluronidase type 1, 60 U/ml DNase I, and 450 U/ml collagenase type I, in 2.5 ml of PBS) to obtain single-cell suspensions. Macrophages were sorted using anti-F4/80 antibody from single-cell suspensions, and then subjected for immunofluorescence analysis.

**1.15 Animal experiments and atherosclerosis analysis**

APOE^-/-^ mice were purchased from Nanjing Model Animal Research Center and housed in the Laboratory Animals Center of the first affiliated hospital Zhejiang university school of medicine, with controlled temperature and humidity. All experimental procedures were approved by the Animal Care Ethics Committee of the Zhejiang University. APOE^-/-^ mice were fed a high fat high cholesterol diet (Western Diet) for 12 weeks to induce atherosclerosis. Thirty ApoE^−/−^ mice were randomly divided into three groups (ten mice per group): normal ApoE^−/−^ mice fed with normal diet group (Control), APOE^-/-^ mice fed with a high fat high cholesterol diet and injected with recombinant SARS-CoV-2 S2 protein group (S2), and APOE^-/-^ mice fed with a high fat high cholesterol diet and injected with PBS group (PBS). Recombinant SARS-CoV-2 S2 protein (5 μg/kg) or PBS (as control) was tail vein injected every three days. 12 weeks later, the mice were anesthetized with sodium pentobarbital (50 mg/kg, i.p.; BHD, Canada). After exposing the abdominal cavity and rapid opening of the right atrium, 5 ml of saline was slowly injected into the left ventricle along the apex of the heart to wash the blood. Next, 4% polyformaldehyde was slowly injected to fix the vascular tissue morphology for 30 min. Thereafter, the aorta and perivascular adipose tissue were removed under a stereomicroscope. The pulmonary arteriovenous malformation was excised, and then the artery together with the heart was removed, followed by placement in 4% paraformaldehyde at room temperature overnight. The aortas were dissected, and whole aortas were opened longitudinally from the aortic arch to the iliac bifurcation, mounted en face, and stained for lipids with Oil Red O (Jiancheng, China). The hearts were embedded in OCT (Tissue-Tek; Sakura, Torrance, CA), and serial 5-mm-thick cryosections from the aortic sinus were mounted and stained with Oil Red O and hematoxylin-eosin. Image analysis was performed by a trained observer blindly to the genotype of the mice. Representative images were obtained, and lesion areas were quantified with Image J. The lesion area in the aorta en face preparations was expressed as a percent of the aortic surface area.

**1.16 Statistical analysis**

Data are presented as the mean ± standard deviation (SD). The differences between two groups were compared by t-test. The differences among multiply groups were compared using ANOVA analysis. A P value of< 0.05 was considered statistically significant and all experiments were repeated at least three times.

**2．Supplementary and more detailed Results**

As letter described, we analyzed recently published single-cell RNA-seq data of peripheral blood mononuclear cells (PBMCs) from COVID-19 patients, and healthy donors, and found that PBMCs from healthy donors were divided into 6 clusters (Figure S1C and S1D), whereas PBMCs from COVID-19 patients were divided into 14 clusters (Figure S1A and S1B). Furthermore, we analyzed the expression levels of lipid uptake-related genes, including CD36, MSR1, and LOX1, and cholesterol efflux-related genes, including ABCA1 and ABCG1, in the PBMCs from COVID-19 patients and healthy donors. As shown in Figure S1E and S1F, CD36 and MSR1 were low expressed in the macrophages (cluster 4) from healthy donors, whereas CD36 and MSR1 were abundantly expressed in the macrophages (cluster 5) from COVID-19 patients. LOX-1 was barely expressed in nearly all kinds of cells from COVID-19 patients. ABCA1 and ABCG1 were nearly expressed in all kinds of cells from COVID-19 patients, and the expression levels of ABCA1 and ABCG1 from COVID-19 patients were comparable to those from healthy donors. The above analysis indicates that SARS-CoV-2 infection might affect CD36 and MSR1 expressions in the peripheral blood macrophages.

As letter described, we constructed atherosclerosis mice model using ApoE-/- mice fed with western diet (WD). Recombinant viral S2 protein (Figure S5A) or PBS (as control) was injected by tail vein every three days. And, mice with S2 protein injection had no obvious effect on weight loss (Figure S5B) and pro-inflammatory factors secretion (Figure S5C). After 12 weeks of S2 injection, we found that recombinant viral S2 protein injection does not obviously affect mice liver and kidney function (Figure S5D-G).


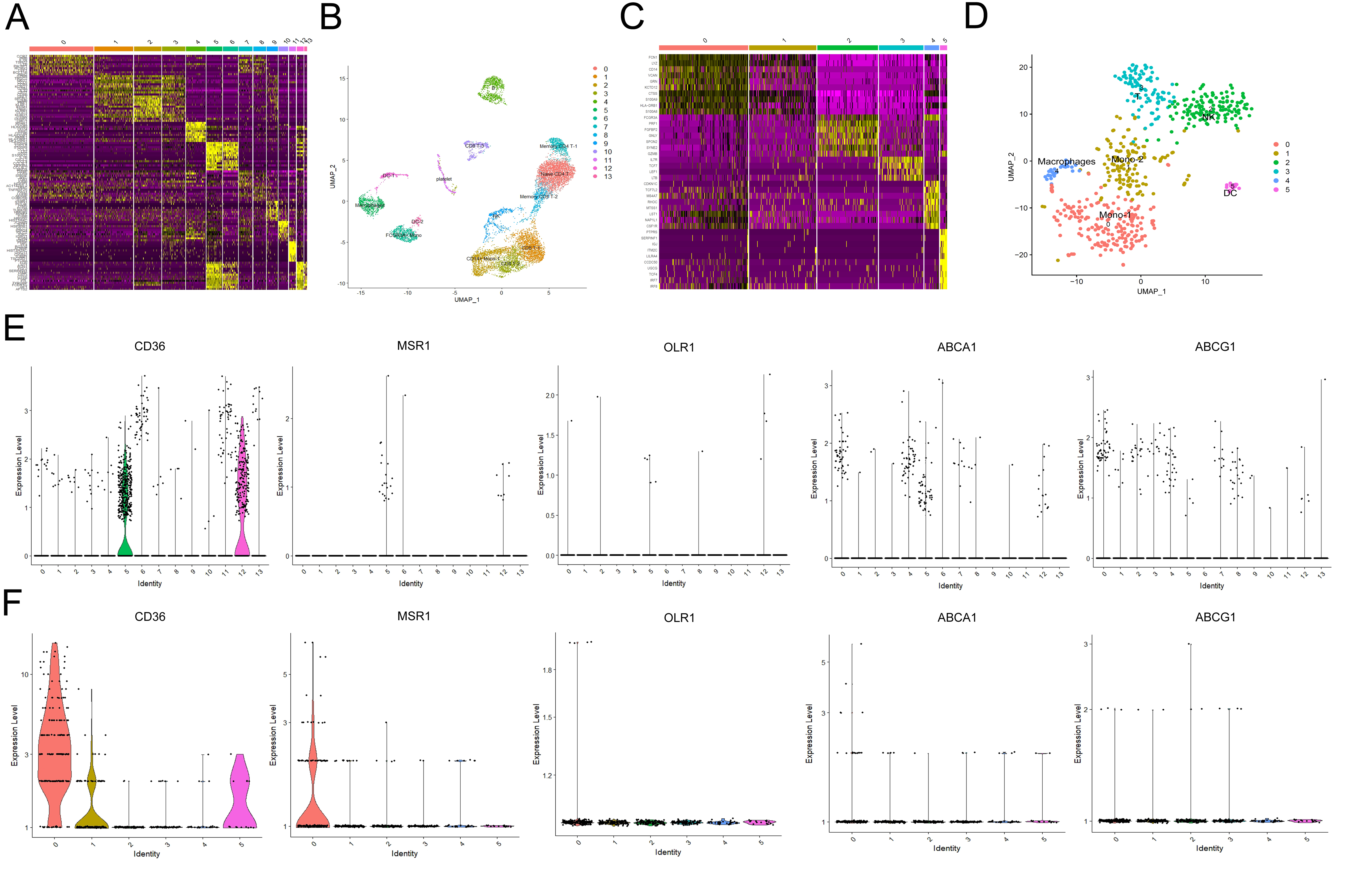


**Supplementary Fig. S1.** SARS-CoV-2 infection might affect CD36 and MSR1 expressions in the peripheral blood macrophages. **(A)** Heatmap analysis of differential gene expressions in all clusters of peripheral blood mononuclear cells (PBMCs) from COVID-19 patients (GSE150861). **(B)** 2D visualization of single-cell clusters (GSE150861) by tSNE. **(C)** Heatmap analysis of differential gene expressions in all clusters of PBMCs from healthy donors. **(D)** 2D visualization of single-cell clusters by tSNE. **(E-F)** An overview of PBMCs from COVID-19 patients (E) or healthy donors (F) at the single-cell level. CD36, MSR1, OLR1, ABCA1 and ABCG1 genes expression for each cell type were visualized using violin plots.


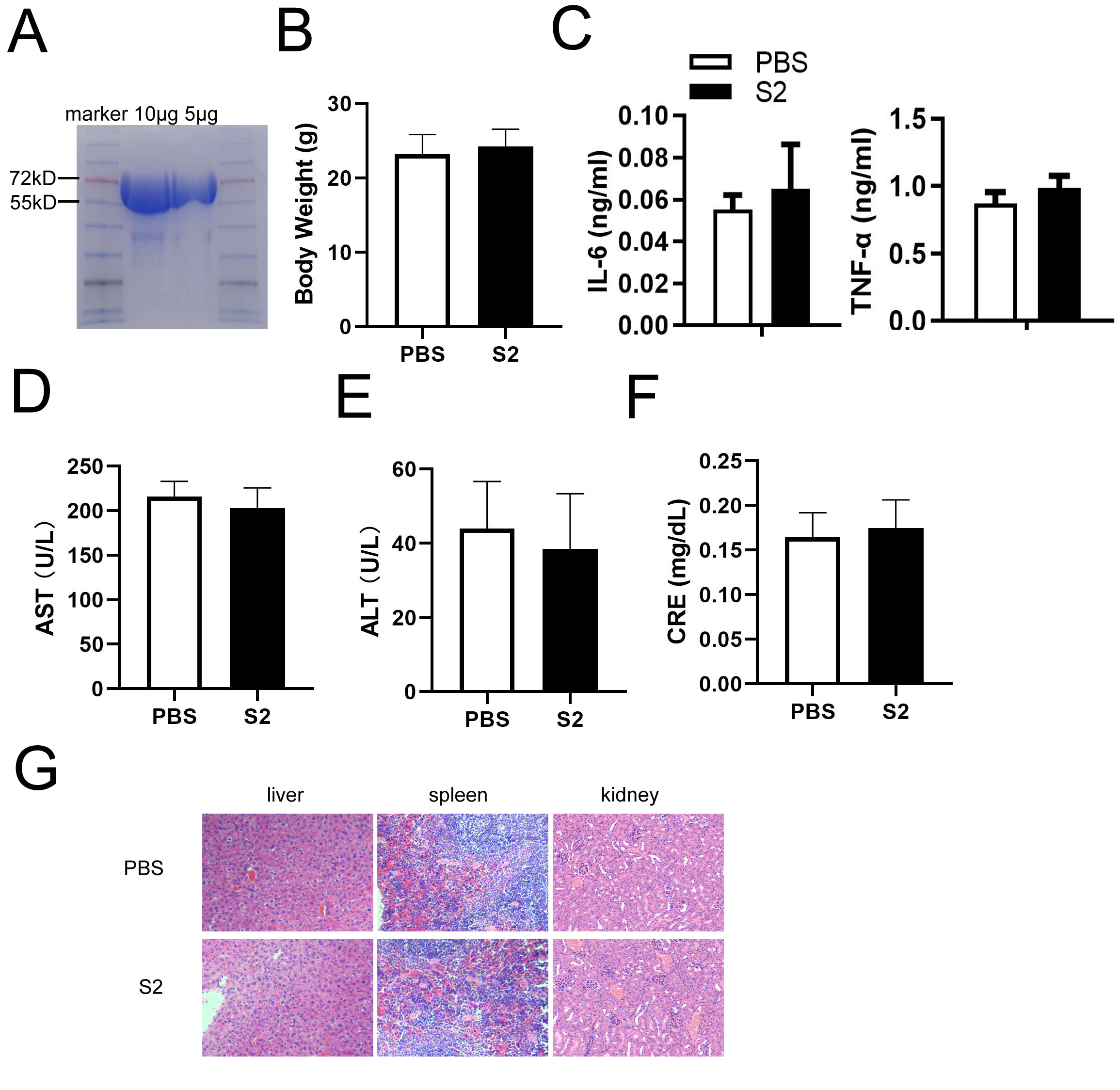


**Supplementary Fig. S2** S2 protein injection has no effect on mice liver and kidney function. **(A)** Coomassie blue staining of recombinant protein HIS tagged S2. **(B)** Body weight analysis of the effects PBS and recombinant SARS-CoV-2 S2 injection on ApoE^-/-^ mice (n=10 per group). **(C-F)** IL-6, TNF-α, AST, ALT and CRE were detected in serum samples of mice treated with PBS or S2. **(G)**: Representative pictures of the HE-stained liver kidney and spleen tissues from PBS or S2 treated mice.
